# HGF Secreted by Activated Kupffer Cells Induces Apoptosis of *Plasmodium*-Infected Hepatocytes

**DOI:** 10.3389/fimmu.2017.00090

**Published:** 2017-02-06

**Authors:** Lígia Antunes Gonçalves, Joana Rodo, Lurdes Rodrigues-Duarte, Luciana Vieira de Moraes, Carlos Penha-Gonçalves

**Affiliations:** ^1^Instituto Gulbenkian de Ciência, Oeiras, Portugal

**Keywords:** *Plasmodium*, Kupffer cells, hepatocytes, HGF, apoptosis, malaria, liver

## Abstract

Malaria liver stage infection is an obligatory parasite development step and represents a population bottleneck in *Plasmodium* infections, providing an advantageous target for blocking parasite cycle progression. Parasite development inside hepatocytes implies a gross cellular insult evoking innate host responses to counteract intra-hepatocytic infection. Using primary hepatocyte cultures, we investigated the role of Kupffer cell-derived hepatocyte growth factor (HGF) in malaria liver stage infection. We found that Kupffer cells from *Plasmodium*-infected livers produced high levels of HGF, which trigger apoptosis of infected hepatocytes through a mitochondrial-independent apoptosis pathway. HGF action in infected hepatocyte primary cultures results in a potent reduction of parasite yield by specifically sensitizing hepatocytes carrying established parasite exo-erythrocytic forms to undergo apoptosis. This apoptosis mechanism is distinct from cell death that is spontaneously induced in infected cultures and is governed by Fas signaling modulation through a mitochondrial-dependent apoptosis pathway. This work indicates that HGF and Fas signaling pathways are part of an orchestrated host apoptosis response that occurs during malaria liver stage infection, decreasing the success of infection of individual hepatocytes. Our results raise the hypothesis that paracrine signals derived from Kupffer cell activation are implicated in directing death of hepatocytes infected with the malaria parasite.

## Introduction

Malaria remains a major global health problem. It is estimated that 3.2 billion people are at risk of developing the disease (1), which represents a serious socioeconomic burden in malaria endemic countries (2). The natural infection is initiated by *Plasmodium* sporozoites that reach the liver and infect hepatocytes—the preferred site of parasite replication and an obligatory step for cycle progression in the mammalian host (3). Through a high-efficacy process of pre-erythrocytic schizogony, one sporozoite develops into a liver stage form that undergoes exponential growth entrenching thousands of merozoites inside the parasitophorous vacuole in each infected hepatocyte (4). Parasite invasion and the expansion of exo-erythrocytic forms (EEFs) inside hepatocytes represent a prominent cellular insult that plausibly elicits host strategies to counteract EEFs development.

Malaria liver stage infection has shown to induce local innate immune responses. *Plasmodium* infection has been shown to trigger a type I interferon response that mediates an inflammatory reaction in the liver (5, 6). *Plasmodium* RNA was shown to work as a ligand of the cytosolic pattern recognition receptor Mda5, which activates a type I IFN response *via* the adaptor molecule Mavs and the transcription factors Irf3 and Irf7 (6). This response decreases liver parasite load possibly because type I IFN signaling in myeloid cells contributes to the recruitment of immune cells to the inflammatory foci around infected hepatocytes (6). In fact, recruitment of natural killer T (NKT) cells in sporozoites-infected livers was shown to be dependent on the expression of interferon-alpha/beta receptor alpha chain (5). Another line of evidence indicates that activation of Kupffer cells is a key component in the outcome of malaria liver stage infection. We have shown that pro-inflammatory activation of Kupffer cells by sporozoites is critically regulated by the innate immunity receptor triggering receptor expressed in myeloid cells 2 (TREM2). The level of TREM2 expression in Kupffer cells directly correlates with natural resistance liver stage infection and with reduced hepatocyte parasite yield (7). Nevertheless, the molecular mechanisms by which Kupffer cells control hepatocyte infection are not completely unveiled.

The outcome of liver stage infection results from balancing host defense responses and parasite mechanisms that counteract such responses. In particular, the success of parasite development and growth inside individual hepatocytes is dependent on the ability to sustain host cell survival until late stages of infection (8). In fact, it has been shown that parasite survival depends on inhibition of hepatocyte apoptosis mechanisms, including p53 downregulation and inhibition of mitochondrial-dependent induced cell death (9, 10).

Once sporozoites cross the hepatic sinusoidal barrier, they traverse several hepatocytes before invading and establishing an intracellular infection (11). Shifting to the invasive mode requires the action of host factors released during hepatocyte traversing (12). Hepatocyte growth factor (HGF) signaling has been shown to facilitate *Plasmodium berghei* sporozoite infection in hepatoma cells (13), possibly by conferring resistance to induced cell death (14). Nevertheless, experimental infection of different hepatoma cell lines showed that the induction of HGF signaling did not improve *Plasmodium falciparum* invasion (15) and was not crucial for infection by *P. falciparum* or *Plasmodium yoelii* (16).

We tested the hypothesis that the local innate immune response induced by *P. berghei* liver stage infection was involved in triggering apoptosis of infected hepatocytes. In particular, we investigated whether HGF signaling pathway is involved in apoptosis of infected hepatocytes and takes part in the response of liver innate immune cells to malaria infection. Our results indicate that activation of Kupffer cells by malaria parasites entails the production of HGF that specifically induce apoptosis of infected hepatocytes. This work supports the view that HGF plays multiple roles during malaria liver stage infection.

## Materials and Methods

### Mice

All procedures involving laboratory mice were in accordance with national (Portaria 1005/92) and European regulations (European Directive 86/609/CEE) on animal experimentation and were approved by the Instituto Gulbenkian de Ciência Ethics Committee, and the Direcção-Geral de Veterinária (the Official National Entity for regulation of laboratory animals usage). The mice were bred and maintained in conventional housing facilities at the Instituto Gulbenkian de Ciência. All experiments were conducted using male mice with 8–15 weeks of age from strains C57BL/6, BALB/c, and B6. lpr (B6.MRL-*Fas^lpr^*/J).

### Hepatocyte Primary Cultures

Mouse primary hepatocytes were prepared as previously described (17). In short, mouse liver lobes were perfused with liver perfusion medium and liver digest medium (Gibco; Invitrogen) at 37°C. Dissociated cells from the tissue were separated using a 1.12, 1.08, and 1.06 g/ml Percoll (GE Healthcare) gradient. Hepatocytes were collected from the gradient solution and cultured in William’s E complete medium (Gibco; Invitrogen) in plates or glass coverslips coated with 0.2% gelatine. The viability of hepatocytes in each experiment preparation was assessed by vital staining with Trypan blue and by cell morphology in FACS analysis. All the experiments were performed with viability of hepatocytes preparations above 80% purity. The remaining dead cells were removed 12 h post-plating (12 h prior infection), as in freshly isolated preparations dead hepatocytes do not attach. HGF conditioning was performed by adding 100 ng/ml of recombinant mouse HGF (eBioscience) 1 h prior infection, with a subsequent wash before infection.

### Parasites and Infection

Green fluorescent protein (*gfp*)-expressing *P. berghei* ANKA (Pb) (18), *P. yoelii* 17XNL (Py), and *Pb-spect(-)1* (19) sporozoites were obtained after dissection of infected salivary glands from *Anopheles stephensi* mosquitoes bred in the insectarium of the Instituto de Medicina Molecular, Lisbon. Briefly, mosquitoes were immobilized, and the thorax and abdomen were separated with fine forceps. The salivary glands were dissected from the thoraces into RPMI medium, collected to a 1.5 ml Eppendorf, and smashed to release the sporozoites. The sporozoites were collected from the supernatant upon a 2 min × 5 min 800 rpm centrifugation (20). The same procedure was applied to non-infected mosquitoes to obtain salivary glands suspensions. Mice were intravenously inoculated with 10^4^ sporozoites each. Hepatocytes were infected with 1.5 to 3.5 × 10^4^ sporozoites, and non-infected controls were mock infected with salivary glands suspensions of non-infected mosquitoes.

### Isolation of Liver Non-Parenchymal Cells (NPCs)

Non-parenchymal cells were isolated from mouse livers at 40 h post-infection. Cells were obtained by adapting a method previously described (21). Briefly, liver lobes were removed and perfused with liver perfusion medium (Gibco; Invitrogen) with 750 mg/l of Collagenase H (Roche) at 37°C. The resulting suspension was filtered through a 100 µm cell strainer (BD Falcon). The dissociated cells were suspended in liver perfusion medium and centrifuged for 10 min at 1,500 rpm, resuspended in RPMI complete medium (Gibco; Invitrogen), mixed in Percoll (GE Healthcare) solution to give a final concentration of 30% Percoll, and then centrifuged at 2,000 rpm for 10 min. The cell pellet was resuspended in RPMI and carefully laid on 30% Percoll solution and centrifuged at 2,000 rpm for 10 min. The cell pellet collected was washed and resuspended in ACK (NH_4_Cl 0.15 M, KHCO_3_ 10 mM, Na_2_EDTA⋅2H_2_O 0.1 mM and pH 7.2) for 3 min to lyse remaining erythrocytes. Cells were washed and centrifuged at 800 rpm for 20 s to discard the remaining hepatocytes; the supernatant was recovered, and NPCs were collected at 1,500 rpm for 5 min.

### Total RNA and cDNA Synthesis

Livers and NPCs were collected at 40 h post-infection, immediately homogenized in denaturing solution and total RNA was obtained using High Pure RNA Tissue Kit and High Pure RNA Kit (Roche). One microgram of total RNA was converted to cDNA (Transcriptor First Strand cDNA Synthesis Kit; Roche). Hepatocytes in culture were collected at 24 h post-infection, the lysis and reverse transcriptase reaction were performed using TaqMan Gene Expression Cell-to-CT kit (Ambion).

### *Plasmodium* RNA Quantification and Hepatocyte Gene Expression

cDNA specific to *P. berghei* 18S rRNA was amplified by using the Taqman specific primers: IGC-Pb1 (forward) 5′-CCGATAACGAACGAGATCTTAACCT-3′, IGC-Pb2 (reverse) 5′-CGTCAAAACCAATCTCCCAATAAAGG-3′ and IGC-Pb3 (specific fluorogenic probe) 5′-FAM–ACTCGCCGCTAATTAG–MGB-3′ (7). cDNA specific to *P. yoelii* 18S rRNA was amplified by using the SYBR green system and the specific primers NYU-Py3 5′-GGGGATTGGTTTTGACGTTTTTGCG-3′ and NYU-Py5 5′-AAGCATTAAATAAA GCGAATACATCCTTAT-3′ (22). *Hgf, Bid, Casp3, Hif*α, *Pik3r1, Cflar, Fadd*, and *Casp8* expression was quantified using TaqMan Gene Expression Assays from ABI (Mm01135185_m1, Mm00432073_m1, Mm01195084_m1, Mm00468872_m1, Mm01282780, Mm01255576_m1, Mm00438861_m1, and Mm00802247_m1, respectively). Endogenous control *Gapdh* (Mouse GAPD Endogenous Control; ABI) was used in multiplex PCR with target genes. PCR reactions were performed with ABI Prism 7900HT, and relative quantification was calculated by the ΔΔCt method, using the mean of the control group as calibrator.

### Hepatocyte Immunocytochemistry: TUNEL, EEFs, and FasL

Hepatocytes cultured on coverslips were fixed 24 or 40 h post-infection with 4% paraformaldehyde (PFA) and incubated with 50 nM NH_4_Cl to extinguish PFA activity. Cells were permeabilized and blocked with blocking solution for 1 h [3% of Bovine Serum Albumin (Calbiochem), 100 mM of glycine, 0.1% of saponin, and 10% of Fetal Calf Serum (Gibco; Invitrogen) in PBS 1×] and stained for apoptosis by Terminal Deoxynucleotidyl Transferase dUTP Nick End Labeling (TUNEL) using “*In Situ* Cell Death Detection—TMR” (Roche); to stain EEFs coverslips were then incubated 45 min at room temperature with blocking solution containing anti-GFP IgG Alexa-488 conjugated antibody (Invitrogen); followed by DAPI staining (Invitrogen) and mounted with MOWIOL (Calbiochem); to stain FasL, cells were incubated 45 min at room temperature with blocking solution containing anti-FasL (CD178.1 Kay10; BD Pharmingen) followed by DAPI staining (Invitrogen) and mounted with MOWIOL (Calbiochem). Cell culture images were acquired using an automated Nikon Eclipse TE2000-S inverted fluorescence microscope, with a 20× objective, covering the total coverslip area. ImageJ (NIH) software was used to differentially identify TUNEL-positive or -negative (live) cells, EEFs and FasL in each coverslip, and to estimate single EEFs area. Results are expressed as the number of TUNEL-positive cells and live cells with EEFs per 100 microscopic fields, or FasL^+^ cells per 10^3^ cells.

### Kupffer Cells FACS Analysis and Purification

Liver macrophages were stained, at 4°C, by incubating liver NPCs with F4/80 APC (A3-1; Serotec), CD11c PE (HL3; BD Pharmingen), and CD49b Biotin (DX5 and streptavidin-PerCP; BD Pharmigen) antibodies. Subsequently, a two-step HGF intracellular staining (H170; Santa Cruz and goat anti-rabbit IgG-FITC) was performed by using the BD Cytofix/Cytoperm kit. FACScalibur cytofluorometer (Becton Dickinson) and FlowJo software were used for FACS analysis. Purified Kupffer cells were obtained through high-speed cell sorting (FACSAria; Becton Dickinson), after staining with F4/80 APC (Serotec).

### Kupffer Cell–Hepatocyte Transwell Cultures

We used a 0.4-µm transwell culture system (Millipore) to allow purified Kupffer cells (upper chamber) to exchange soluble factors during 12 h with primary hepatocyte cultures (lower chamber), before infection. Hepatocytes were then infected with *P. berghei* ANKA sporozoites and fixed for parasite and apoptosis staining or collected for RNA analysis at 24 h post-infection. For HGF/MET signaling blocking, we added anti-Met antibodies (N-17; Santa Cruz Biotechnology) at 400 ng/ml when purified Kupffer cells were put in culture with primary hepatocytes followed by an additional 400 ng/ml added 1 h prior infection.

### HGF ELISA

Hepatocyte growth factor secretion in the supernatants from transwell cultures was evaluated by sandwich ELISA. Briefly, maxisorp plates were coated with a capture anti-HGF antibody (H-170;, Santa Cruz) in 0.5 M Carbonate Bicarbonate Solution. Detection used anti-HGF-biotinylated antibody (BAF2207; R&D). Samples were quantified against a standard curve with mouse recombinant HGF (eBioscience).

### Fas Pathway Modulation

Fas-blocking experiments were performed by using either a blocking antibody or a pan-caspase inhibitor. We used 10 µg/ml of anti-FasL antibody (CD178.1 Kay10; BD Biosciences) and 20 µM of Z-VAD-FMK (pan-caspase inhibitor – 550377; BD Biosciences), added 1 h prior infection and using the appropriate controls. Fas signaling was induced with 1 µg/ml of anti-Fas antibody (CD95 Jo2; BD Biosciences) 18 h post-infection with *P. berghei*. *Cflar* knockdown was achieved through a mixture of three distinct siRNAs (s63907, s63908, and s63909; Ambion), at a concentration of 20 µM that was delivered to primary hepatocytes with Oligofectamine (Invitrogen). Control samples included untransfected cells and cells transfected with a negative control siRNA (Ambion) or with a positive control targeting mouse *Gapdh* (Ambion).

### Statistical Analysis

All experiments were performed with a minimum of triplicate samples, and data are representative of at least three independent experiments. Results are presented as average values and error bars represent SD. Statistical analysis used a Mann–Whitney test, and *p* < 0.05 was considered statistically significant.

## Results

### HGF Impacts on the Yield of *Plasmodium* Liver Stage Infection

**Figure 1:**
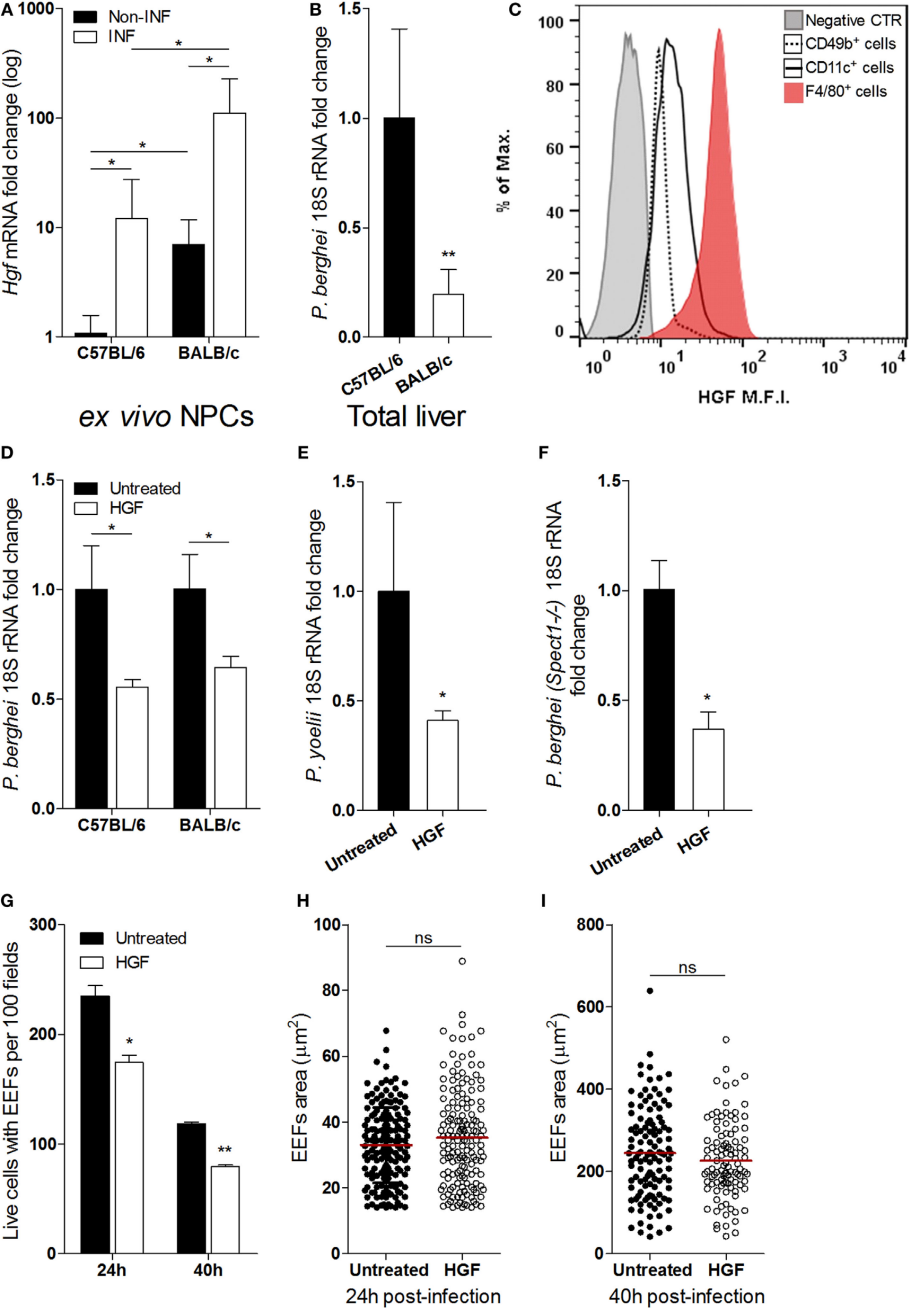
**Hepatocyte growth factor (HGF) and yield of malaria liver stage infection *in vivo* and *in vitro***. Differential resistance to malaria liver stage infection in C57/BL/6 and BALB/c mice correlates with increase of HGF expression in non-parenchymal cells (NPCs) **(A,B)**: **(A)** *Hgf* mRNA quantification in liver NPCs preparations of infected and non-infected mice and **(B)** liver parasite burden measured by qRT-PCR of *Plasmodium berghei* 18S rRNA, measured at 40 h p.i. with *P. berghei* sporozoites. FACS analysis of liver NPCs isolated from C57BL/6-infected mice revealed high HGF intracellular expression in F4/80^+^ cells **(C)**. HGF pre-treatment of hepatocyte primary cultures infected with *P. berghei* ANKA reduced malaria liver stage parasite yield in C57BL/6 and BALB/c hepatocytes **(D)** and in C57BL/6 hepatocytes infected with *Plasmodium yoelii* **(E)** or *Spect1* deficient *P. berghei* **(F)**, at 24 h p.i. Effect of HGF pre-treatment in the counts of viable hepatocytes containing exo-erythrocytic forms (EEFs) **(G)** and EEFs size **(H,I)**, at 24 and 40 h p.i. in C57BL/6 hepatocyte primary cultures (non-parametric Mann–Whitney test, *p < 0.05 and **p ≤ 0.001). Data are represented as mean ± SD of three livers per mouse strain or triplicate cultures that were performed in at least three independent experiments.

Liver NPCs, specifically Kupffer cells, have a role in the natural control of *Plasmodium* liver stage expansion (7). We found that *Hgf* mRNA expression in isolated non-parenchymal liver cells was highly increased upon *in vivo* infection with *P. berghei* sporozoites (Figure 1A) and correlated with relative resistance to parasite liver stage expansion in BALB/c as compared to C57BL/6 mice (Figure 1B). It has been reported that HGF and its mRNA localize exclusively to NPCs in the liver (23–25), and we have confirmed that expression of *Hgf* mRNA in primary hepatocytes was not detectable, irrespective to infection with *P. berghei* (data not shown). Given that HGF has been shown to facilitate liver stage infection (13, 14, 16), we investigated the cellular source of HGF in infected livers. FACS analysis of NPCs isolated from sporozoite-infected mice revealed that F4/80^+^ cells, representing Kupffer cells, expressed intracellular HGF as opposed to other NPCs, e.g., CD11c^+^ (dendritic cells) or CD49b^+^ cells that include NKT and NK cells (Figure 1C). Furthermore, we found that depletion of liver macrophages abrogated the augmented expression of HGF in the liver induced by infection (Figure S1 in Supplementary Material) suggesting that liver macrophages are the main *in vivo* source of HGF in malaria liver stage infection. To untangle the effect of HGF in *Plasmodium* liver stage infection, we pre-treated hepatocyte primary cultures of C57BL/6 and BALB/c mice with HGF 1 h prior infection and observed that HGF conditioning similarly impairs parasite yield in both mouse strains (Figure 1D). Moreover, HGF pre-treatment of C57BL/6 hepatocytes infected with *P. yoelii* (Figure 1E) or *P. berghei-spect(-)1* (Figure 1F) revealed that reduction of parasite yield induced by HGF transcends *Plasmodium* species and is independent of hepatocyte traversing (19). In addition, we found that reduction of parasite burden by HGF was attributable to a significant decrease in the number of hepatocytes carrying EEFs (Figure 1G), detectable from 24 h post-infection onward. In contrast, HGF conditioning was not associated with altered EEFs size, neither at earlier stages (24 h) (Figure 1H) nor at the end of the liver infection (40 h) (Figure 1I). These results show that HGF conditioning reduces the number of hepatocytes that carry EEFs, rather than affecting its development suggesting that Kupffer cell-derived HGF impacts on the survival of infected hepatocytes.

### HGF Promotes Apoptosis of EEF-Carrying Hepatocytes

**Figure 2:**
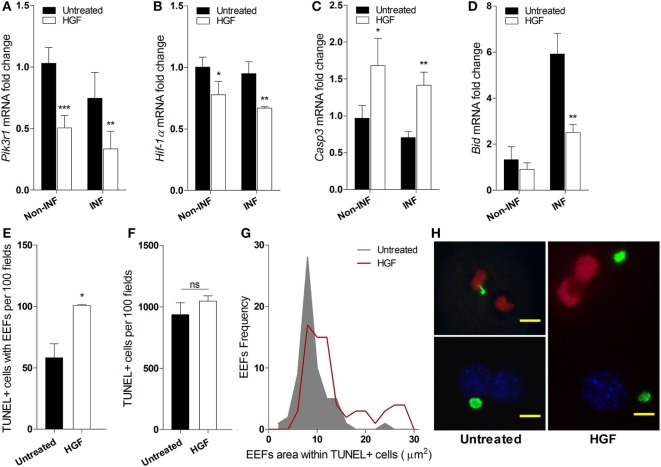
**Hepatocyte growth factor (HGF)-conditioning targets exo-erythrocytic form (EEF)-containing hepatocytes for mitochondrial-independent apoptosis**. Effect of HGF on the mRNA expression of selected apoptosis-related markers *Pik3r1* **(A)**, *Hif-*α **(B)**, *Caspase 3* **(C)**, and *Bid* **(D)** in infected (INF) and non-infected (non-INF) hepatocyte cultures at 24 h p.i. Results represent qRT-PCR estimates relative to non-infected untreated hepatocyte cultures. Effects of HGF pre-treatment on the viability of primary hepatocytes at 24 h p.i. with *Plasmodium berghei* sporozoites: **(E)** apoptotic cells containing EEFs, **(F)** total apoptotic cells (TUNEL; TUNEL-positive), and **(G)** size distribution of EEFs harbored in apoptotic cells. **(H)** Representative immunofluorescence images of primary hepatocyte cultures in the presence (right) or absence (left) of HGF pre-treatment, evidencing larger EEFs in TUNEL + cells after HGF pre-treatment (TUNEL-positive nuclei in red, DAPI-positive in blue and EEFs in green; scale bar 10 µm) (non-parametric Mann–Whitney test, *p < 0.05, **p ≤ 0.001, and ***p ≤ 0.0001). Data are represented as mean ± SD of triplicate cultures that were performed in at least three independent experiments.

We investigated whether the reduced number of developed EEFs upon HGF pre-treatment was due to apoptosis of infected hepatocytes. Analysis of mRNA expression of key informative genes showed that HGF induces downregulation of PI3K a component of the HGF/c-Met pathway that is involved in apoptosis protection (26, 27) (Figure 2A). Moreover, HGF downregulated *Hif1*-alpha that has been associated with hepatocyte apoptosis and efficiency of *P. berghei* infection (28, 29) (Figure 2B). Furthermore, upregulation of *Caspase 3* (Figure 2C), with a concomitant decrease of *Bid* expression (Figure 2D), suggests that HGF acts through a mitochondrial-independent apoptosis pathway in response to hepatocyte *Plasmodium* infection (30).

Interestingly, we observed that HGF pre-treatment specifically increased apoptosis susceptibility in parasitized hepatocytes but did not affect bulk apoptosis in the infected cultures (Figures 2E,F), as detected by TUNEL assays. Remarkably, in HGF pre-treated cultures, the size distribution of EEFs inside apoptotic hepatocytes significantly deviated to higher values (*p*-value <0.0001) (Figure 2G). Thus, in untreated infected cultures, hepatocytes carrying small EEFs were the major target of spontaneous apoptosis, but under HGF conditioning significant apoptosis was observed in infected hepatocytes with larger EEFs (Figures 2G,H). Collectively, these data indicate that parasitized primary hepatocytes respond to HGF treatment through a mitochondrial-independent apoptosis mechanism, which operates in hepatocytes carrying early liver schizont stages that are refractory to spontaneous apoptosis.

### Kupffer Cell-Derived HGF and Efficiency of Primary Hepatocyte Infection

**Figure 3:**
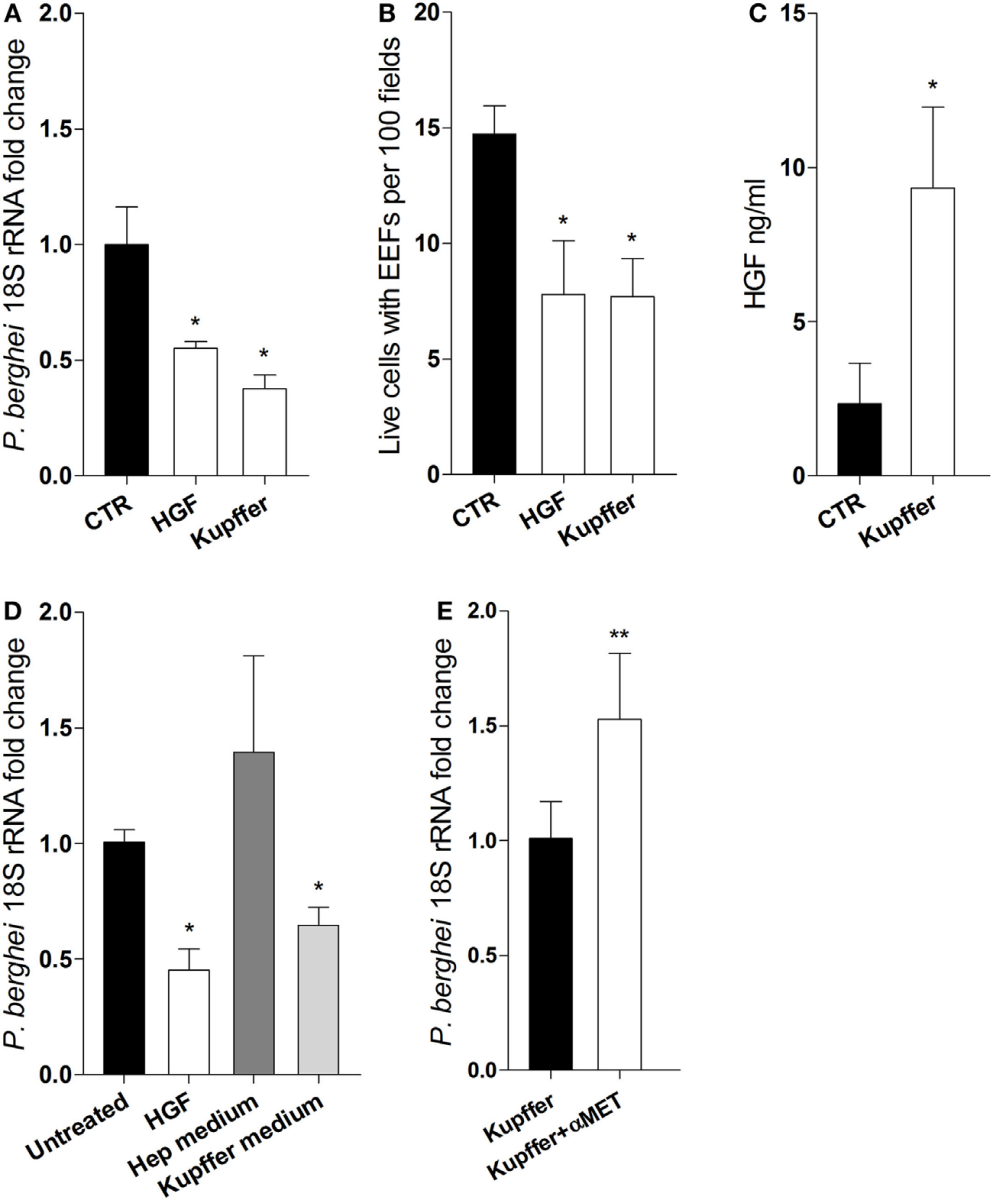
**Kupffer cells secrete hepatocyte growth factor (HGF) and control efficacy of primary hepatocyte infection**. Transwell co-cultures of liver F4/80^+^ cells (upper chamber) and primary hepatocytes (lower chamber) were compared at 24 h p.i. (Kupffer), with single hepatocytes cultures pre-treated with HGF (HGF) or left untreated (CTR), for parasite yield **(A)** and number of viable hepatocytes containing exo-erythrocytic forms (EEFs) **(B)**. Secreted HGF was evaluated in the supernatant of untreated hepatocytes and co-cultures after infection **(C)**. Parasite yield in hepatocyte cultures treated with HGF or incubated with culture supernatant from previous single hepatocytes culture (Hep medium) or from previous hepatocyte-F4/80^+^ cells co-culture in transwell (Kupffer medium) **(D)**. Parasite yield at 24 h p.i. in transwell co-cultures of liver F4/80^+^ cells and primary hepatocytes in the absence (Kupffer) or presence (Kupffer + αMET) of anti-MET blocking antibody **(E)** (non-parametric Mann–Whitney test, *p < 0.05 and **p ≤ 0.001). Data are represented as mean ± SD of triplicate cultures that were performed in at least three independent experiments, except **(E)** that represents cumulative data of two independent experiments with triplicate samples.

We ask whether HGF secreted by Kupffer cells could impair hepatocyte infection *in vitro*. We performed transwell co-culture experiments using FACS sorted F4/80^+^ cells from infected livers and naïve primary hepatocytes. Remarkably, we observed that parasite yield and the number of infected hepatocytes were reduced in the presence of Kupffer cells, to an extent comparable to the cultures pre-treated with HGF (Figures 3A,B), indicating that this effect is attributable to soluble factors derived from Kupffer cells. Moreover, HGF was detected in the supernatants of Kupffer cells/hepatocyte cultures (Figure 3C), which were able to reduce parasite yield when used as media in pure hepatocyte cultures (Figure 3D). Furthermore, adding anti-MET antibodies in the co-cultures to block the action of Kupffer cells-derived HGF led to increased parasite yield in hepatocytes (Figure 3E). Altogether, these results strongly suggest that HGF is secreted by Kupffer cells and acts in an endocrine fashion to reduce successful progression of infection in parasitized hepatocytes.

### Involvement of Fas-Mediated Apoptosis in *Plasmodium* Liver Stage Infection

**Figure 4:**
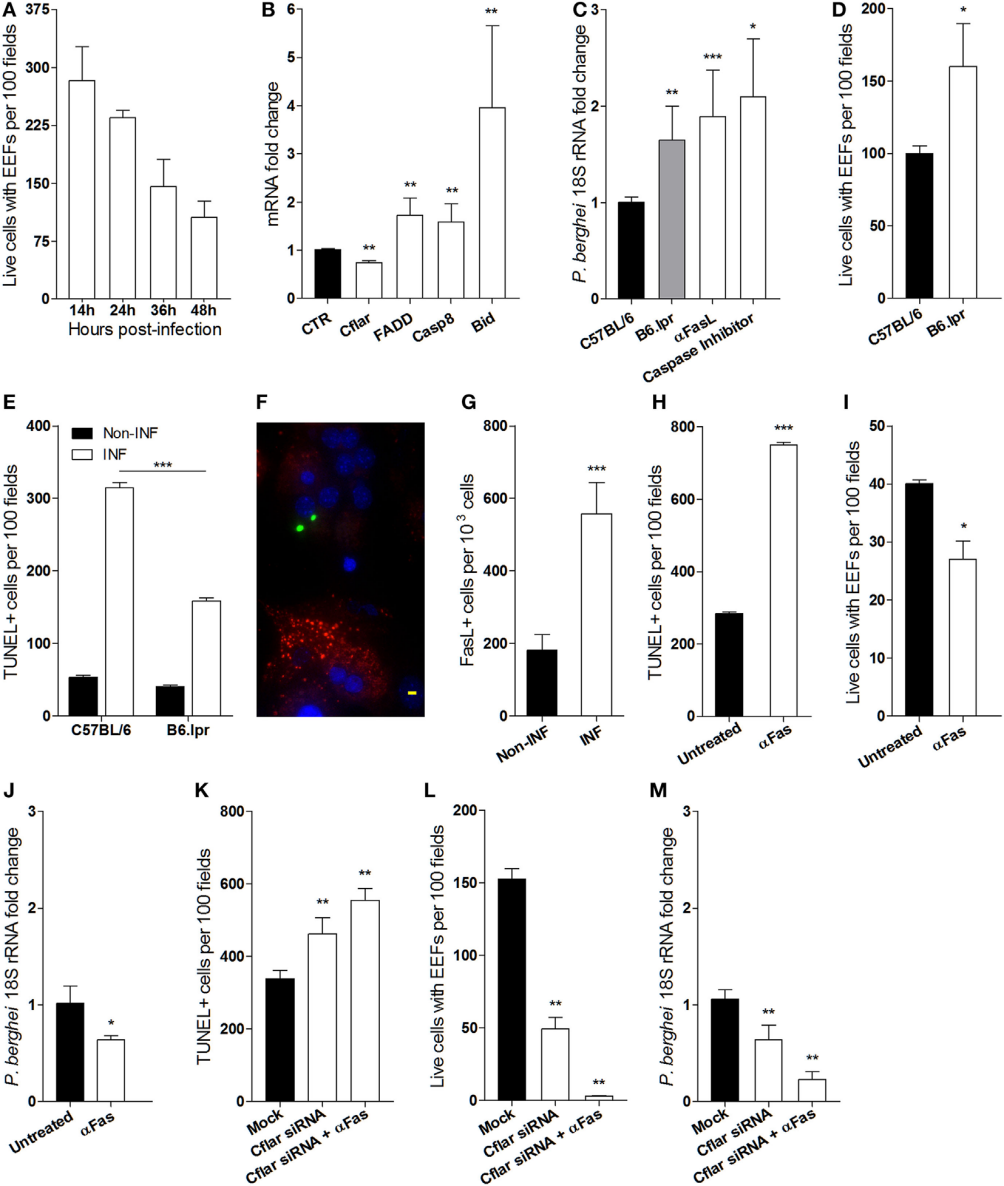
**Fas-mediated hepatocyte apoptosis impairs the success of *Plasmodium berghei* infection (A) exo-erythrocytic form (EEF)-containing viable hepatocytes were counted at indicated times in infected primary cultures**. **(B)** mRNA quantification of Fas pathway genes at 24 h p.i., relative to non-infected control (CTR) hepatocyte cultures. **(C)** Parasite yield in infected cultures of Fas-deficient hepatocytes from B6. *lpr* mice or after blocking Fas pathway with anti-FasL antibody was measured by qRT-PCR of *P. berghei* 18S rRNA at 40 h p.i. **(D)** EEF-containing viable hepatocytes of B6. *lpr* and C57BL/6 mice were counted and **(E)** the number of apoptotic cells was determined by TUNEL staining in infected and non-infected cultures at 24 h p.i. **(F)** Representative immunofluorescence image of non-infected hepatocyte expressing FasL (red) in the vicinity of *P. berghei*-gfp (green) infected hepatocytes [nuclei stained with DAPI (blue)], scale bar—20 µm and **(G)** counting of FasL-expressing cells in infected and non-infected cultures at 24 h p.i. **(H,K)** number of apoptotic cells (TUNEL-positive), **(I,L)** number of viable hepatocytes containing EEFs, and **(J,M)** parasite yield were measured in primary hepatocyte cultures in the presence or absence of signaling by anti-Fas antibody and/or *Cflar* siRNA (non-parametric Mann–Whitney test, *p < 0.05, **p ≤ 0.001, and ***p ≤ 0.0001). Data are represented as mean ± SD of triplicate cultures that were performed in at least three independent experiments.

Additionally, it has been described that the failure of EEFs to attain full development *in vitro* is attributable to an hepatocyte autonomous mitochondrial-dependent apoptosis response (10). In line with these observations, we asked whether reduction of the number of EEFs in infected primary hepatocyte cultures occurs as well in the absence of HGF. To ascertain the success of *P. berghei* sporozoites infection in hepatocyte primary cultures, we performed time course counting of infected hepatocytes, which showed that less than 50% of the initial infected cells attained EEF full development (Figure 4A). To investigate whether the aborted EEFs were related to apoptosis induction in *P. berghei*-infected cultures, we analyzed the expression of apoptosis genes in hepatocyte primary cultures and found out that Fas pathway signaling-related genes were significantly altered at 24 h p.i. A strong upregulation of *Bid* coupled with a slight upregulation of other proapoptotic genes and downregulation of the anti-apoptotic *Cflar*, represent a signature that Fas pathway signaling was induced in infected primary cultures (Figure 4B), presumably executing a Type II apoptotic response that requires mitochondrial participation (31). To test whether Fas pathway signaling was detrimental to successful hepatocyte infection, we analyzed primary hepatocytes from *lpr* mice (Fas-null mutant), which demonstrated that at 24 h p.i. the parasite yield was significantly increased in the absence of Fas pathway signaling. An effect that was also observed by blocking Fas pathway signaling with anti-Fas Ligand antibodies or with a pan-Caspase inhibitor (Figure 4C). Furthermore, the absence of Fas pathway signaling improved the number of successfully infected hepatocytes (Figure 4D), possibly because apoptosis induced by infection was partially dependent on Fas pathway signaling (Figure 4E). Interestingly, immunofluorescence microscopy of infected cultures revealed prominent FasL expression in non-infected hepatocytes as compared to infected hepatocytes (Figure 4F), indicating that induction of FasL expression by *P. berghei* infection (Figure 4G) impacts the viability of both infected and non-infected hepatocytes. Conversely, enhancing Fas pathway signaling in hepatocytes either by adding anti-Fas antibodies or by knocking down *Cflar*, a potent Fas pathway inhibitor (32), increased apoptosis susceptibility in infected hepatocyte cultures (Figures 4H,K). In these conditions, we observed decreased number of hepatocytes containing EEFs (Figures 4I,L) and reduced parasite yield (Figures 4J,M). The effects of *Cflar* knockdown were exacerbated by anti-Fas stimulation (Figures 4K–M) and strongly suggest that *Cflar* inhibition sensitized hepatocytes to undergo apoptosis through the Fas pathway. These experiments provide evidence that apoptosis signals evoked by the parasite favor Fas-dependent hepatocyte apoptosis in infected cultures leading to a decrease in the number of infected primary hepatocytes that support full parasite development.

## Discussion

This work revealed an unpredicted hepatocyte—Kupffer cell crosstalk mediated by HGF, which sensitizes *Plasmodium*-infected hepatocytes to undergo apoptosis. Furthermore, we provide evidence that Fas-mediated hepatocyte apoptosis is spontaneously induced in infected cultures. Together, the results support the notion that hepatocyte response to *Plasmodium in vitro* infection entails two distinct apoptosis mechanisms: (1) an HGF-stimulated mitochondrial-independent response that preferably targets hepatocytes harboring early liver schizonts and (2) a Fas-mediated mitochondrial-dependent apoptosis mechanism, acting on both infected and non-infected hepatocytes. Our results suggest that these two responses operate at distinct phases of hepatocyte infection and use different genetic programs for apoptosis induction, representing diverse modes to control malaria liver stage infection.

We have previously shown that expression of innate immunity receptors in Kupffer cells, namely, TREM2, is involved in the genetic resistance to malaria liver stage infection in mouse models (7). We now demonstrate an additional mechanism of parasite yield control mediated by Kupffer cells that specifically targets infected hepatocytes in culture. After *in vivo* infection, HGF is upregulated in the liver and is mainly expressed by Kupffer cells. This is in line with the notion that HGF and its mRNA localize exclusively to non-parenchymal liver cells (23), namely, Kupffer cells (24). Furthermore, increased HGF mRNA expression in the liver is accompanied by a rise in circulating HGF (33), suggesting an endocrine mechanism of action. Expression of HGF by Kupffer cells has been observed in the context of liver repair and regeneration (34), and our *in vitro* data indicate that HGF also acts on parasitized hepatocytes to induce apoptosis. These observations are in line with a dual role of HGF in promoting either hepatocyte apoptosis or proliferation depending on the contextual cues (28, 34, 35). In fact, it was reported that HGF signaling in hepatoma cell lines favors sporozoite transition to the invasive mode and cell survival upon *Plasmodium* sporozoite infection, in a species-specific fashion (13, 14, 16). However, our data imply that HGF also has a role in controlling infection by inducing apoptosis of parasitized hepatocytes through a mechanism that transcends *Plasmodium* species and does not require parasite traversing. Our results embody the hypothesis that HGF produced by infection-activated Kupffer cells, sensitizes primary hepatocytes to engage a mitochondrial-independent apoptosis program (Figures 2A–D) in parasitized cells with established EEFs (Figures 2E–H), potentially overcoming parasite strategies to block spontaneous apoptosis (9).

On the other hand, we observed that infected hepatocyte cultures exhibited spontaneous apoptosis, possibly explaining the reduction of infected cells number in the course of *in vitro* infection. In line with previous reports (10), we found that inhibition of Fas pathway significantly decreased apoptosis in infected cultures while increasing parasite yield, suggesting that susceptibility of infected hepatocytes to Fas-mediated apoptosis is an efficient way to control initial parasite liver stage infection. Coherently, stimulation of signaling through the Fas pathway decreased the number of infected hepatocytes that supported successful infection. Intriguingly, FasL appears to be overproduced by non-infected hepatocytes that surround individual infected hepatocytes, suggesting that FasL-expressing cells provide a paracrine signal to induce apoptosis of the infected cells (Figure 4). Although susceptibility to Fas-mediated apoptosis plays a role in reducing parasite yield, this effect appeared to affect equally infected and non-infected hepatocytes in the infected cultures. In contrast, HGF signaling promotes a shift from mitochondrial-dependent to mitochondrial-independent apoptosis (30) and preferentially targets infected cells in primary cultures. It remains to be investigated what are the intracellular requirements that license apoptosis by each of the two apoptosis mechanisms in order to disentangle their role in the time course of hepatocyte infection.

The decision of whether one infected hepatocyte undergoes apoptosis or support full parasite development might rely on time windows of host apoptosis induction, which is counteracted by effective parasite apoptosis-inhibiting activity (9). In fact, treatment with proapoptotic drugs *in vivo* was shown to delay or abrogate transition to blood stage infection (36). We have noted that the size of the EEFs found in TUNEL-positive cells in non-sensitized cultures represent early forms of liver stage parasite, suggesting that spontaneous hepatocyte apoptosis is able to deter parasite development at early stages. In contrast, HGF treatment was capable of inducing apoptosis in hepatocytes containing EEFs of larger sizes, indicating that this apoptosis pathway is effective at later stages of parasite development. Together, these observations indicate that the fate of infected hepatocytes is dictated by paracrine interactions occurring between Kupffer cells and hepatocytes that direct hepatocyte apoptosis. Manipulation of such apoptosis mechanisms may provide an effective tool to block or suppress liver stage progression and confer disease protection.

The malaria liver stage infection is a current target for identification of protective responses based on the killing activity of parasite-specific CD8 T cells (37) operating independently of bone marrow-derived antigen-presenting cells (APCs) (38). The mechanisms of apoptosis of infected hepatocytes here described have potential impact in building up immunological memory against parasite epitopes presented by MHC class I molecules. On the one hand, phagocytosis of infected hepatocytes apoptotic bodies by professional APCs may represent an additional route to prime CD8 T cells specific for malaria liver stage epitopes (39). On the other hand, responses that increase apoptosis resistance of infected hepatocytes may provide an opportunity for enhancing antigen presentation in the liver, with consequent activation of liver-resident CD8 T cells that prevent transition to blood stage infection.

## Author Contributions

LG and CP-G conceived the study and wrote the manuscript; LG designed, performed, analyzed, and interpreted data from all the experiments; JR, LR-D, and LM participated in the design, acquisition, analysis, and interpretation of data relative to FACs and ELISA experiments; CP-G interpreted the data. All the authors critically revised and approved the version to be published.

## Conflict of Interest Statement

The authors declare that the research was conducted in the absence of any commercial or financial relationships that could be construed as a potential conflict of interest.

## Abbreviations

HGF, hepatocyte growth factor; KC, Kupffer cell; EEFs, exo-erythrocytic forms; TREM2, triggering receptor expressed in myeloid cells 2; NKT, natural killer T; ACK, ammonium chloride potassium; TUNEL, terminal deoxynucleotidyl transferase dUTP nick end labeling; Ct, cycle threshold; DAPI, 4′,6-diamidino-2-phenylindole; NPCs, liver non-parenchymal cells; lpr, lymphoproliferation; FasL, Fas ligand.
