# Supplementary material for: HGF Secreted by Activated Kupffer Cells Induces Apoptosis of Plasmodium-Infected Hepatocytes
Source: Front Immunol. 2017 Feb 6;8:90. doi: 10.3389/fimmu.2017.00090 (PMC5292919; doi:10.3389/fimmu.2017.00090)
Supplement: Supplementary file 1 [file presentation_1.pdf]

Supplementary Figure S1

HGF secreted by Activated Kupffer Cells Induces Apoptosis of Plasmodium-Infected Hepatocytes

Lígia Antunes Gonçalves, Joana Rodo, Lurdes Rodrigues-Duarte, Luciana Vieira de Moraes and Carlos Penha-Gonçalves

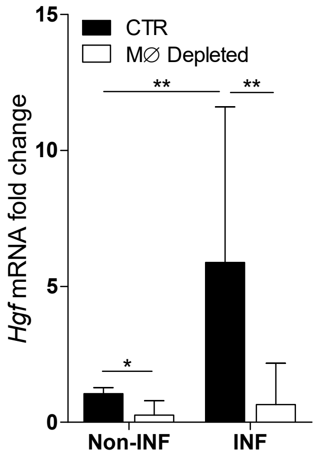

Depletion of liver macrophages decreases *Hgf* mRNA expression. Macrophages depleted (M∅ depleted) *in vivo* by clodronate-containing liposomes show reduced *Hgf* mRNA expression in total livers of non-infected (Non-INF) and infected (INF) mice, and is evidenced in the INF group. *Hgf* mRNA expression was measured by qRT-PCR in six livers per mouse group, all from C57BL/6 mouse strain. (Non-parametric Mann-Whitney test, $p<0.05$ (*) and $p\leq0.001$ (**))
